# Supplementary material for: Spinal fluid IgG antibodies from patients with demyelinating diseases bind multiple sclerosis-associated bacteria
Source: J Mol Med (Berl). 2021 Jun 8;99(10):1399–411. doi: 10.1007/s00109-021-02085-z (PMC8185491; doi:10.1007/s00109-021-02085-z)
Supplement: Supplementary file 1 — (DOCX 385 kb) [file 109_2021_2085_MOESM1_ESM.docx]

**Table S1. Control and commercial antibodies used for the study.**

1. **Control antibody preparations used for indirect ELISA**

| **Antibody** | **Application** | **Vendor** | **Catalog number** | **Clonality** | **Host** | **Isotype** |
| --- | --- | --- | --- | --- | --- | --- |
| Anti-*E. coli* lipopolysaccharide | primary | Abcam | Ab35654 | Monoclonal | mouse | IgG_2b_ |
| Anti-*Pseudomonas aeruginosa* | primary | ThermoFisher | PA1-73116 | Polyclonal | Rabbit | IgG |
| Anti-bacterial Peptidoglycan | primary | EMD Millipore | MAB-995 | Monoclonal | Mouse | IgG_1_ |
| HRP anti-mouse IgG | secondary | Vector | PI-2000 | - | Horse | - |
| HRP anti-human IgG | secondary | Jackson Immuno | 309-035-082 | - | Rabbit | - |
| HRP anti-Rabbit IgG | secondary | Vector | PI-1000 | - | Goat | - |

1. **Dilutions of primary and secondary antibodies used for indirect ELISA.** The strong positive (++) was titrated to an OD of ~1.0 and the weak positive (+) to just above background. Background was determined in wells containing no primary antibody, secondary antibody only.

| **Organism** | **Control Primary Ab** | **Strong + Control**  **Dilution** | **Weak + Control Dilution** | **Anti-Rabbit Secondary Ab Dilution** | **Experimental Human CSF Samples** | **Secondary Anti-Human Ab Dilution** |
| --- | --- | --- | --- | --- | --- | --- |
| Akkermansia | PA1-73116 | 400 | 6,400 | 3,000 | neat | 10,000 |
| Lactobacillus | PA1-73116 | 200 | 1600 | 3,000 | neat | 10,000 |
| Pseudomonas | PA1-73116 | 6,400 | 102,400 | 3,000 | neat | 10,000 |
| Atopobium | PA1-73116 | 400 | 6,400 | 3,000 | neat | 10,000 |
| Bacteroides | PA1-73116 | 200 | 800 | 3,000 | neat | 10,000 |
| Odoribacter | PA1-73116 | 400 | 3,200 | 3,000 | neat | 10,000 |
| Cutibacterium | PA1-73116 | 400 | 3,200 | 6,000 | neat | 20,000 |
| Streptococcus | PA1-73116 | 200 | 400 | 6,000 | neat | 20,000 |
| Porphyromonas | PA1-73116 | 400 | 102,400 | 6,000 | neat | 20,000 |
| Fusobacterium | PA1-73116 | 1,600 | 64,000 | 6,000 | neat | 20,000 |
