# Supplementary material for: Spinal fluid IgG antibodies from patients with demyelinating diseases bind multiple sclerosis-associated bacteria
Source: J Mol Med (Berl). 2021 Jun 8;99(10):1399–411. doi: 10.1007/s00109-021-02085-z (PMC8185491; doi:10.1007/s00109-021-02085-z)
Supplement: Supplementary file 2 — (DOCX 18 kb) [file 109_2021_2085_MOESM2_ESM.docx]

**Table S2. Statistical Analysis**

1. **Summary of CSF serologic reactivity and statistical comparisons between the groups.**

| Organism | DD Group^1^ | OND Group^1^ | Control Group^1^ | ANOVA^2^ | DD v Control^3^ | OND v Control^3^ |
| --- | --- | --- | --- | --- | --- | --- |
| *Akkermansia muciniphila* | 3.94 ± .67 | 3.90 ± .48 | 1.59 ± .35 | p = 0.005 | P = 0.002 | P = 0.003 |
| *Atopobium vaginae* | 4.73 ± .72 | 4.44 ± .61 | 2.01 ± .47 | P = 0.006 | P = 0.003 | P = 0.007 |
| *Bacteroides fragilis* | 2.28 ± .29 | 3.09 ± .57 | 1.35 ± .15 | P = 0.003 | P = 0.006 | P = 0.006 |
| *Lactobacillus paracasei* | 4.51 ± .91 | 4.54 ± .98 | 2.08 ± .44 | P = 0.049 | P = 0.029 | P = 0.027 |
| *Odoribacter splanchnicus* | 2.73 ± .38 | 3.20 ± .81 | 1.78 ± .31 | P = 0.105 | P = .049 | P = .105 |
| *Pseudomonas aeruginosa* | 3.67 ± .65 | 3.65 ± .52 | 2.21 ± .37 | P = 0.101 | P = 0.080 | P = 0.028 |
| *Cutibacterium acnes* | 5.55 ± .29 | 6.02 ± .42 | 4.92 ± .62 | P = 0.360 | P = NS | P = NS |
| *Fusobacterium necrophorum* | 1.16 ± .12 | 1.41 ± .11 | 0.90 ± .10 | P = 0.024 | P = .032 | P = .010 |
| *Porphyromonas gingivalis* | 1.62 ± .19 | 1.82 ± .23 | 1.57 ± .17 | P = 0.715 | P = NS | P = NS |
| *Streptococcus mutans* | 2.23 ± .22 | 2.34 ± .38 | 1.82 ± .23 | P = 0.335 | P = NS | P = NS |

^1^ Mean Elisa Index values ± standard error of the mean

^2^ One-way ANOVA, unweighted analysis

^3^ Mann-Whitney nonparametric testing, 2-tailed

1. **Linear regression of EI, the assay types and subject groups.**

|  | Residual Degrees  of Freedom | Residual Deviance | Degrees of Freedom | Deviance | F | P-value |
| --- | --- | --- | --- | --- | --- | --- |
| Age, Sex | 323 | 171 | - | - | - | - |
| Age, Sex, and Organism | 314 | 124 | 9 | 46.16 | 17.67 | <0.0001 |
| Age, Sex, Organism, and Group | 312 | 94 | 2 | 30.45 | 52.46 | <0.0001 |
| Age, Sex, Organism, Group, and Interaction Term | 294 | 85 | 18 | 8.62 | 1.65 | 0.0478 |
