# Supplementary material for: Spinal fluid IgG antibodies from patients with demyelinating diseases bind multiple sclerosis-associated bacteria
Source: J Mol Med (Berl). 2021 Jun 8;99(10):1399–411. doi: 10.1007/s00109-021-02085-z (PMC8185491; doi:10.1007/s00109-021-02085-z)
Supplement: Supplementary file 3 — (DOCX 21 kb) [file 109_2021_2085_MOESM3_ESM.docx]

**Table S3. CSF serologic responses to the 10 MS candidate bacteria in the DD group.** Indirect ELISA was performed on CSF from 14 subjects with definite demyelinating disease. Commercial human CSF (Randox Laboratories Ltd, UK; ([IgG] 10 mg/dl) was used as the positive control. IgG-depleted commercial human CSF (Randox Laboratories Ltd, UK) was utilized as the negative (calibration) control. The EI of the negative control is defined as 1.0.

| Subject | Neurologic Diagnosis | Ocb | BBB Intact | Akk | Atop | Bact | Lacto | Odor | Pseudo | Cuti | Fuso | Porphy | Strep |
| --- | --- | --- | --- | --- | --- | --- | --- | --- | --- | --- | --- | --- | --- |
| DD-03 | ADEM | Positive  3 Bands | No | 10.6 | 12.1 | 5.2 | 11.6 | 1.9 | 8.6 | 4.2 | 0.6 | 2.9 | 2.7 |
| DD-08 | RRMS | Positive  5 Bands | Yes | 2.3 | 3.7 | 1.8 | 1.6 | 1.8 | 2.6 | 6.2 | qns | qns | qns |
| DD-10 | Progressive MS | Positive  3 Bands | Yes | 5.4 | 5.3 | 2.2 | 1.8 | 1.9 | 4.3 | 6.3 | 1.6 | 2.0 | 2.9 |
| DD-11 | MS untyped | Negative | nd | 1.5 | 2.2 | 1.3 | 1.3 | 1.7 | 2.0 | qns | qns | qns | qns |
| DD-13 | ADEM | Negative | No | 3.7 | 6.8 | 1.8 | 5.0 | 1.8 | 9.2 | qns | qns | qns | qns |
| DD-17 | Tumefactive MS | Negative Matched | nd | 3.3 | 4.5 | 1.2 | 1.3 | 1.2 | 2.4 | 4.3 | 1.0 | 0.9 | 1.8 |
| DD-19 | MS Untyped | Negative  1 Band | nd | 2.2 | 1.6 | 1.0 | 3.1 | 2.8 | 1.2 | 6.7 | 1.0 | 0.9 | 1.9 |
| DD-21 | CIS | Negative | nd | 4.9 | 4.2 | 2.4 | 3.1 | 4.7 | 3.1 | 5.2 | 2.0 | 2.4 | 1.8 |
| DD-71 | RRMS | Positive  6 Bands | No | 4.7 | 7.3 | 3.1 | 6.2 | 2.7 | 4.8 | 3.8 | 1.4 | 1.7 | 0.6 |
| DD-72 | RRMS | Negative  Matched | Yes | 7.1 | 4.3 | 2.6 | 4.1 | 5.8 | 3.8 | 5.3 | 1.3 | 1.4 | 2.9 |
| DD-79 | Rhombencephalitis | Positive  4 Bands | No | 1.7 | 3.8 | 1.3 | 1.7 | 1.5 | 1.8 | 5.6 | 1.1 | 1.2 | 2.2 |
| DD-80 | Tumefactive MS | Negative | No | 2.2 | 3.7 | 2.8 | 8.2 | 2.4 | 2.3 | 6.6 | 1.0 | 1.4 | 2.7 |
| DD-82 | Anti-MOG | Negative Matched | Yes | 3.4 | 4.7 | 3.1 | 10.4 | 4.7 | 3.8 | 6.7 | 1.0 | 1.6 | 3.1 |
| DD-83 | Tumefactive MS | Positive  6 Bands | Yes | 2.2 | 2.1 | 2.3 | 3.8 | 3.4 | 1.5 | 5.6 | 0.8 | 1.4 | 2.0 |
| Positive Control | - | - | - | 6.6 | 9.2 | 5.2 | 9.3 | 5.4 | 7.3 | 4.0 | 1.9 | 3.4 | 4.1 |

EI = ELISA Index Value: EI ≤ 1.0 = negative EI 1.1 – 2.9 = weak positive EI 3.0 – 4.9 = positive EI ≥ 5.0 = strong positive

DD = demyelinating disease, CIS = clinically isolated syndrome, ADEM = acute disseminated encephalomyelitis,

RRMS = relapsing-remitting multiple sclerosis, OCB = results of clinical oligoclonal band testing,

BBB = blood-brain barrier intactness, based on the albumin index value where normal (0-9) is intact, > 9 is compromised,

ND = not tested qns = quantity not sufficient for testing

Akk = *Akkermansia muciniphila*; Lacto = *Lactobacillus paracasei*; Pseudo = *Pseudomonas aeruginosa*; Atop = *Atopobium vaginae*; Bact = *Bacteroides fragilis*; Odor = *Odoribacter splanchnicus*; Strep = *Streptococcus mutans*; Cuti = *Cutibacterium acnes*; Porphy = *Porphyromonas gingivalis*; Fuso = *Fusobacterium necrophorum*
