# Supplementary material for: Spinal fluid IgG antibodies from patients with demyelinating diseases bind multiple sclerosis-associated bacteria
Source: J Mol Med (Berl). 2021 Jun 8;99(10):1399–411. doi: 10.1007/s00109-021-02085-z (PMC8185491; doi:10.1007/s00109-021-02085-z)
Supplement: Supplementary file 4 — (DOCX 23 kb) [file 109_2021_2085_MOESM4_ESM.docx]

**Table S4. CSF serologic responses to the 10 MS candidate bacteria in the OND and control groups.** Indirect ELISA was performed on CSF from 8 subjects with other neurologic diseases (OND) and on CSF from 13 control subjects. Commercial human CSF (Randox Laboratories Ltd, UK; ([IgG] 10 mg/dl) was used as the positive control. IgG-depleted commercial human CSF (Randox Laboratories Ltd, UK) was utilized as the negative (calibration) control. The EI of the negative control is defined as 1.0.

| Subject | Neurologic Diagnosis | OCB | BBB Intact | Akk | Atop | Bact | Lacto | Odor | Pseudo | Cuti | Fuso | Porphy | Strep |
| --- | --- | --- | --- | --- | --- | --- | --- | --- | --- | --- | --- | --- | --- |
| OND-63 | Migraine, Spina Bifida | ND | ND | 2.8 | 3.3 | 1.0 | 1.1 | 1.0 | 2.9 | 4.1 | 1.5 | 1.2 | 1.1 |
| OND-64 | Microvascular Disease | Negative  Matched | Yes | 4.9 | 6.8 | 6.1 | 7.8 | qns | 5.8 | qns | qns | qns | qns |
| OND-73 | Atypical Stroke | Negative | No | 4.1 | 4.4 | 2.9 | 8.5 | 3.4 | 5.5 | 5.8 | 1.6 | 1.5 | 2.7 |
| OND-75 | CSF Shunt Infection | ND | ND | 3.3 | 2.4 | 1.7 | 2.9 | 2.0 | 2.9 | 7.0 | 1.5 | 2.0 | 1.6 |
| OND-76 | Pudendal Neuralgia | Negative | Yes | 2.0 | 2.3 | 2.0 | 1.3 | 1.2 | 1.3 | 6.1 | 1.0 | 1.7 | 2.1 |
| OND-78 | CNS Vasculitis | Negative | Yes | 3.5 | 4.3 | 3.9 | 4.2 | 2.6 | 3.1 | qns | qns | qns | qns |
| OND-81 | CNS Lyme Disease | Positive  13 Bands | No | 4.0 | 5.6 | 3.1 | 4.5 | 6.2 | 3.8 | 6.5 | 1.1 | 2.8 | 3.6 |
| OND-84 | Neurosyphilis | Negative | No | 6.4 | 6.4 | 3.9 | 6.0 | 6.1 | 4.0 | 6.7 | 1.7 | 1.7 | 3.0 |
| C-23 | Normal Pressure Hydrocephalus | ND | ND | 0.4 | 0.4 | 0.9 | 0.9 | 1.8 | 1.9 | 6.3 | 1.9 | 1.1 | 3.2 |
| C-25 | Pseudotumor Cerebri (Iih) | ND | ND | 0.4 | 0.4 | 2.1 | 1.7 | 2.0 | 2.0 | 7.0 | 0.9 | 1.6 | 1.7 |
| C-27 | Normal Pressure Hydrocephalus | ND | ND | 0.4 | 0.4 | 1.7 | 1.6 | 1.6 | 1.9 | 5.9 | 0.7 | 2.1 | 3.2 |
| C-28 | Pseudotumor Cerebri (Iih) | ND | ND | 0.8 | 1.1 | 0.9 | 1.8 | 0.9 | 1.1 | 5.9 | 0.8 | 1.1 | 1.3 |
| C-30 | Normal Pressure Hydrocephalus | ND | ND | 4.5 | 5.2 | 1.9 | 4.1 | 2.1 | 2.9 | 6.4 | 0.6 | 2.0 | 2.3 |
| C-31 | Normal Pressure Hydrocephalus | ND | ND | 2.9 | 3.1 | 1.9 | 5.7 | 1.8 | 2.5 | 3.5 | 1.2 | 2.0 | 2.3 |
| C-32 | Cranioplasty, Stroke | ND | ND | 2.9 | 3.0 | 1.6 | 2.7 | 4.5 | 5.2 | 7.2 | 0.7 | 1.7 | 1.8 |
| C-33 | Acute Hydrocephalus | ND | ND | 1.0 | 1.5 | 1.0 | 1.0 | 0.7 | 1.1 | qns | 0.8 | 1.2 | 1.2 |
| C-34 | Normal Pressure Hydrocephalus | ND | ND | 2.8 | 1.7 | 1.1 | 1.4 | 1.2 | 3.1 | 2.0 | 0.9 | 2.9 | 1.6 |
| C-58 | Hydrocephalus Due To Tumor Resection | ND | ND | 1.1 | 1.4 | 1.0 | 1.0 | 0.7 | 1.0 | 2.7 | 0.9 | 1.3 | 1.3 |
| C-59 | Hydrocephalus Post Tbi | ND | ND | 1.5 | 5.4 | 2.0 | 4.0 | 3.7 | 1.9 | 5.7 | qns | qns | qns |
| C-67 | Normal Pressure Hydrocephalus | ND | ND | 1.4 | 1.7 | 1.0 | 1.0 | 1.2 | 3.9 | 5.9 | 0.7 | 1.0 | 1.3 |
| C-68 | Hydrocephalus Post Meningitis | ND | ND | 0.6 | 0.8 | 0.5 | 0.4 | 1.1 | 0.4 | 0.6 | 0.7 | 0.9 | 0.6 |
| Positive Control | - | - | - | 6.6 | 9.2 | 5.2 | 9.3 | 5.4 | 7.3 | 4.0 | 1.9 | 3.4 | 4.1 |

EI = ELISA Index Value: EI ≤ 1.0 = negative EI 1.1 – 2.9 = weak positive EI 3.0 – 4.9 = positive EI ≥ 5.0 = strong positive

IIH = Idiopathic Intracranial Hypertension, TBI = Traumatic Brain Injury, OCB = results of clinical oligoclonal band testing,

BBB = blood-brain barrier intactness, based on the albumin index value where normal (0-9) is intact, > 9 is compromised

ND = not tested qns = quantity not sufficient for testing

Akk = *Akkermansia muciniphila*; Lacto = *Lactobacillus paracasei*; Pseudo = *Pseudomonas aeruginosa*; Atop = *Atopobium vaginae*; Bact = *Bacteroides fragilis*; Odor = *Odoribacter splanchnicus*; Strep = *Streptococcus mutans*; Cuti = *Cutibacterium acnes*; Porphy = *Porphyromonas gingivalis*; Fuso = *Fusobacterium necrophorum*
