# Supplementary material for: Spinal fluid IgG antibodies from patients with demyelinating diseases bind multiple sclerosis-associated bacteria
Source: J Mol Med (Berl). 2021 Jun 8;99(10):1399–411. doi: 10.1007/s00109-021-02085-z (PMC8185491; doi:10.1007/s00109-021-02085-z)
Supplement: Supplementary file 5 — (DOCX 26 kb) [file 109_2021_2085_MOESM5_ESM.docx]

**Table S5. MS Microbial Candidate List (abridged).** Unbiased (deep, next generation) RNA sequencing was performed on 18 formalin-fixed paraffin-embedded primary demyelination brain specimens taken from 17 patients (MS Group), 16 epilepsy brain specimens (Control Group), and 2 blanks (no tissue). This list is revised from that previously published because specimens with gram-negative bacterial sequence (from the sequencing reagents) have been eliminated or resequenced.[1] Methods used for RNA extraction, library preparation, sequencing, and analysis are identical to those previously described, except that MAPQ filtering was not performed here.

Criteria for inclusion into this candidate table include:

- Overexpression in the MS group where
  - the MS:Control Ratio ≥ 3.0
  - the MS:Blank Ratio > 1.5
- > 1000 total reads summed from all 17 specimens in the MS group

| UID^1^ | Genus | Sum MS Group Reads^2^ | Sum Control Group Reads^3^ | MS:Control Ratio^4^ | Sum Blank Reads^5^ | MS:Blank Ratio^6^ | # MS Group Specimens where q<0.05^7^ |
| --- | --- | --- | --- | --- | --- | --- | --- |
| 239934 | Akkermansia | *25,348* | *314* | 80.7 | *316* | 80.2 | 1 |
| 1380 | Atopobium | *1,426* | *186* | 7.7 | *108* | 13.2 | 1 |
| 816 | Bacteroides | *23,762* | *1,284* | 18.5 | *10,566* | 2.2 | 2 |
| 1578 | Lactobacillus | *166,088* | *23,846* | 7.0 | *9,936* | 16.7 | 1 |
| 283168 | Odoribacter | *2,070* | *28* | 73.9 | *70* | 29.6 | 3 |
| 545932 | Luz24likevirus^8^ | *8,338* | *0* | inf | *0* | inf | 5 |
| 1743 | Cutibacterium | *549,878* | *140,666* | 3.9 | *311,544* | 1.8 | 0 |
| 848 | Fusobacterium | *29,446* | *2,320* | 12.7 | *416* | 70.8 | 2 |
| 836 | Porphyromonas | *8,574* | *2,188* | 3.9 | *794* | 10.8 | 0 |
| 1301 | Streptococcus | *570,636* | *44,464* | 12.8 | *54,190* | 10.5 | 4 |

^1^ UID = Utah Identification Number (taxon)

^2^ Sum of mapped read pairs in the demyelination (MS) group

^3^ Sum of mapped read pairs in the control group

^4^ (Sum mapped read pairs in the MS group) divided by (Sum mapped read pairs in the Control group)

^5^ Sum of mapped read pairs in the blank specimens

^6^ (Sum mapped read pairs in the MS group) divided by (Sum mapped read pairs in the blanks)

^7^ Number of MS specimens where read-pair mappings to these taxa were significantly increased (q<0.05) compared to the control group

^8^ Luz24likevirus is a Pseudomonas phage.

inf = infinity, denominator is zero
