# Supplementary material for: Spinal fluid IgG antibodies from patients with demyelinating diseases bind multiple sclerosis-associated bacteria
Source: J Mol Med (Berl). 2021 Jun 8;99(10):1399–411. doi: 10.1007/s00109-021-02085-z (PMC8185491; doi:10.1007/s00109-021-02085-z)
Supplement: Supplementary file 6 — (DOCX 21 kb) [file 109_2021_2085_MOESM6_ESM.docx]

**Figure S1. Relationship of ELISA index to blood-brain barrier dysfunction in subjects with demyelinating disease.** This exploratory analysis shows the expected ELISA Index (EI) values for a given albumin index (a marker of BBB intactness) for each bacterial antigen. Experimentally determined EI values were plotted against the clinically determined albumin index values, and linear regression was performed (solid lines with dashed 95% confidence bands).^1^ Normal albumin index values (0-9) are indicated by the yellow boxes. Each data point is labeled with its demyelinating disease subject number. More antibodies are expected to leak across the blood-brain barrier (BBB) as the albumin index value rises. Data points above the expected EI for their given albumin index (e.g. subjects 10 and 72 in the anti-Akkermansia plot) are interpreted as evidence for intrathecal antibody synthesis.

Reference:

1. Ivashchenko, R., T. Bilogurova, I. Bykov, L. Dolgaya, V. Iegorov, T. Malko, and A. Protsenko, *Prism 9 for macOS*. 2020, GraphPad Software, LLC: San Diego, CA.
